# Protein Traffic Is an Intracellular Target in Alcohol Toxicity

**DOI:** 10.3390/ph4050741

**Published:** 2011-05-17

**Authors:** Guillermo Esteban-Pretel, María Pilar Marín, Ana M. Romero, Xavier Ponsoda, Raul Ballestin, Juan J. Canales, Jaime Renau-Piqueras

**Affiliations:** 1 Sección de Biología y Patología Celular, Centro de Investigación, IIS-La Fe, Valencia, Spain; E-Mails: esteban_gui@gva.es (G.E.-P.); marin_mpm@gva.es (M.P.M.); amarope@alumni.uv.es (A.M.R.); renau_jai@gva.es (J.R.-P.); 2 Department of Biología Celular, Universidad de Valencia, Valencia, Spain; E-Mails: Ponsoda@uv.es (X.P.); raul.ballestin@gmail.com (R.B.); 3 Behavioural Neuroscience, Department of Psychology, University of Canterbury, Private Bag 4800, Christchurch 8140, New Zealand; E-Mail: juan.canales@canterbury.ac.nz (J.J.C.)

**Keywords:** ethanol, neurons, astrocytes, intracellular traffic, nucleocytoplasmic transport

## Abstract

Eukaryotic cells comprise a set of organelles, surrounded by membranes with a unique composition, which is maintained by a complex synthesis and transport system. Cells also synthesize the proteins destined for secretion. Together, these processes are known as the secretory pathway or exocytosis. In addition, many molecules can be internalized by cells through a process called endocytosis. Chronic and acute alcohol (ethanol) exposure alters the secretion of different essential products, such as hormones, neurotransmitters and others in a variety of cells, including central nervous system cells. This effect could be due to a range of mechanisms, including alcohol-induced alterations in the different steps involved in intracellular transport, such as glycosylation and vesicular transport along cytoskeleton elements. Moreover, alcohol consumption during pregnancy disrupts developmental processes in the central nervous system. No single mechanism has proved sufficient to account for these effects, and multiple factors are likely involved. One such mechanism indicates that ethanol also perturbs protein trafficking. The purpose of this review is to summarize our understanding of how ethanol exposure alters the trafficking of proteins in different cell systems, especially in central nervous system cells (neurons and astrocytes) in adult and developing brains.

## Contents

1. Introduction

*1.1. Intracellular traffic.*

*1.2. Alcohol Consumption and Development.*

2. Alcohol Affects Glycosylation and the Intracellular Trafficking of Proteins.

*2.1. Alcohol Effects on Protein Secretion by Exocytosis.*

*2.2. Alcohol, GA Morphology and Glycosylation in the Liver and Central Nervous System Cells.*

*2.3. Ethanol Exposure Affects Several Factors Involved in the Transport of Nascent Proteins from the ER to the GA, as well as the Traffic Through this to the TGN.*

*2.4. Effects of Alcohol Exposure on the Cytoskeleton.*

*2.5. Ethanol Affects Endocytosis.*

*2.6. Alcohol-Induced Alterations in Nucleocytoplasmic Shuttle.*

3. Conclusions

4. Funding

5. Acknowledgments

6. References

## 1. Introduction

### 1.1. Intracellular Traffic

One of the main characteristics of eukaryotic cells is the presence of numerous membrane-bound compartments which allow the separation/specialization of processes within the cell. The membranes surrounding these organelles have a unique protein composition; thus this compartmentalization requires cells to transport macromolecules from their synthesis site to the appropriate specialized compartment. With membrane proteins, once they have been synthesized by ribosomes, they are translocated to the endoplasmic reticulum (ER) where these proteins are scrutinized to ensure their correct folding and to undergo a series of modifications, such as initial glycosylation, before being packaged into transport intermediates or vesicles to be then moved forward to the Golgi apparatus (GA) entry site. In this organelle, secretory proteins are then transferred through the GA cisternae to the trans-Golgi network (TGN), or GA exit site [1-4]. During their passage through the various cisternae of the GA, proteins are modified by a large number of glycosidase and glycosyltransferase residents in the GA [5-7]. At the TGN, proteins are sorted according to their final destinations, which can be plasma membrane (PM) insertion or extracellular secretion. The TGN is a highly dynamic compartment involved in the sorting of the cargo for delivery to multiple destinations. Thus, proteins are either packaged into membrane carriers for transport to the apical and basolateral membranes in polarized cells, regulated secretory granules and the endosome/lysosome system, or recycled from the TGN to earlier compartments in the secretory pathway. Therefore, the GA can be considered the central station along the secretory pathway, which also serves for the posttranslational modification of proteins and lipids (mostly by glycosylation). In addition, cell surface proteins, ligands, lipids, and other solutes can be introduced into cells through a mechanism known as endocytosis, which starts with the internalization of these structures and molecules into primary endocytic vesicles.

In both processes (the secretory pathway, or exocytosis, and the endocytosis), the cytoskeleton plays an important role by providing filamentous tracks along which membrane vesicular carriers are transported from one compartment to another. The cytoskeleton of eukaryotic cells is composed of microtubules (MT), actin microfilaments and intermediate filaments (IF) [4].

### 1.2. Alcohol Consumption and Development

Alcohol abuse is linked to the occurrence of many pathological conditions. Over the years, the effects of alcohol on many body organ systems and its role in the development of a variety of medical problems, including liver cirrhosis, cardiovascular diseases, brain damage and fetal abnormalities, have been documented, and it is now accepted that the relation between alcohol consumption and health outcomes is a complex and multidimensional one. Moreover, alcohol has been shown to be causally related to more than 60 different medical conditions [8] [for further information, see *The 10th Special Report to the U.S. Congress on Alcohol & Health* and the following issues of *Alcohol Research & Health*: **1997**, *21* (1); **2003**, *27* (2-4)].

On the other hand, clinical and experimental evidence indicates that alcohol consumption during pregnancy disrupts the developmental processes in the central nervous system, leading to the depression of neurogenesis, delayed and aberrant neuronal migration, and anomalous development [9]. Thus, the offspring of women who drink alcohol during pregnancy may be affected either by alcohol-related or fetal alcohol syndrome (FAS), the commonest preventable causes of mental retardation. However, it is important to note that the effects of prenatal alcohol exposure lie in a continuum of physical anomalies and behavioral and cognitive deficits, at the end of which we find FAS. Therefore, the term fetal alcohol spectrum disorders (FASD) [10,11] has been adopted as a nondiagnostic umbrella term to describe this range of effects. The prevalence of FASD is estimated to be at about 2-5% of all births [12].

No single mechanism has sufficed to account for these varying effects of alcohol on brain development, and multiple factors are likely involved; moreover, a number of mechanisms have been proposed. Thus, there is extensive evidence to support that alcohol affects a variety of cellular processes in the developing brain through different molecular mechanisms [13,14]. Among these mechanisms, ethanol has been proposed to perturb protein trafficking, including protein glycosylation, exocytosis and endocytosis [14,15-20].

This review aims to summarize the general concepts of the effect of alcohol exposure on protein sorting in the secretory and endocytic pathways in different cell systems, especially on neurons and astrocytes in the adult and developing central nervous system. As is well known, brain activity involves continuous interactions between neurons and astrocytes.

## 2. Alcohol Affects Glycosylation and Intracellular Trafficking of Proteins

### 2.1. Alcohol Effects on Protein Secretion by Exocytosis

Different types of cells are able to secrete substances into the extracellular medium with which it may exert different functions such as neurotransmitters, antibodies, hormones, etc. Ethanol exposure alters secretion in a wide variety of cell types, including hepatocytes and HepG2 cells, pancreatic acinar cells, adipocytes, gastric parietal cells, Kupffer cells, alveolar epithelial cells, macrophages, astrocytes and neurons. Studies into this subject indicate that there are various mechanisms through which alcohol may alter by increasing or decreasing cellular secretion, which range from alterations in protein synthesis to alterations in intracellular traffic. In addition, several of these mechanisms can co-exist in the same cell. Thus in hepatocytes, one of the cell types in which the effect of chronic and acute exposure of ethanol on secretion has been better studied, it has been reported that both chronic and acute alcohol exposure depress hepatic protein synthesis *in vivo*, which could be related to an impaired translation of mRNA at the peptide-chain initiation level (for a review, see [21]). In addition, and as indicated below in detail, alcohol also affects other processes involved in hepatocyte secretion by exocytosis, such as glycosylation or intracellular transport. In other cell types on which this review focuses, such as growing astrocytes, it has been demonstrated that ethanol decreases the secretion by exocytosis of important proteins, including nerve growth factor (NGF) and several neural cell adhesion molecule (N-CAM) isoforms [22,23]. However, the mechanisms involved in the retention of these molecules in astrocytes have not yet been clarified. On the other hand, information is available on the effect that alcohol has on neuron secretion. Thus, ethanol exposure has been reported to alter the secretion of several neurotrophins, such as brain-derived neurotrophic factor (BDNF), and neurotrophin-3 in neonatal rat cerebellar granule cells [24]. Moreover, other studies have shown that a diminished secretion of BDNF in alcohol-exposed granule cells could be due to alcohol-induced alterations in its receptor, TrkB, as a result of a decrease in the mRNA for TrkB and TrkB-T1 [25]. Moreover, alcohol-stimulated dopamine release in the nucleus accumbens of rats has been shown [26]. Finally, ethanol has been seen to inhibit the TGFbeta1-induced up-regulation of the N-CAM expression in neuroblastoma cells [27].

### 2.2. Alcohol, GA Morphology and Glycosylation in the Liver and Central Nervous System Cells

Hepatomegaly is common in alcoholics and alcohol-fed experimental animals. It is closely associated with increased hepatocellular protein levels that contribute to hepatocyte hypertrophy or “ballooning”. Pioneering studies performed more than 30 years ago and more recent ones have demonstrated that both chronic and acute alcohol exposure results in the retention of newly synthesized proteins in the GA in ethanol-treated hepatocytes [28-32], suggesting a blockage in the vesicle delivery from the TGN to the basolateral surface. Nonetheless, it has been also indicated that chronic ethanol consumption does not selectively inhibit the synthesis and secretion of specific plasma proteins into the blood [33]. Different authors have taken advantage of this overloading of GA in alcohol-exposed animals to isolate different fractions of this cell component [34]. From that time to the present-day, many studies have aimed to elucidate the mechanisms involved in this retention, and a consensus has been reached, although it still remains to be clarified, about this ethanol-induced retention of proteins being a very constant effect, irrespectively of the model used [30]. As the transport of nascent proteins from the ER to the GA, and the traffic through this to the TGN, are very complex processes involving several steps and structures, alcohol has been proposed to possibly act to affect one of these steps, or more. Therefore, before analyzing the possible targets of alcohol on the traffic of proteins from the ER to the TGN, we wish to briefly discuss the most remarkable steps of this process [see 1-4]. As mentioned above, this traffic is performed using the vesicles formed at donor organelles through the action of several distinct coat proteins, including clathrin, COPI and COPII, each of which participates in specific pathways. Some, like clathrin, require the participation of adaptor proteins to bind to the vesicle membrane. COPII participates in anterograde transport from the ER to the cis-GA network, whereas COPI is involved in the retrograde transport from this network back to the ER. The disassembly of the coat, whose components are recycled to their place of origin, probably exposes the targeting machinery on the vesicle surface (i.e., the SNAREs) for the subsequent delivery of the cargo to the appropriate organelle. SNARE proteins, which participate in basic fusion machinery, comprise two distinct families of integral membrane proteins. One type of SNARE, the v-SNARE, is found on the vesicle membrane, while members of the second family, t-SNAREs, are mainly found on the target membrane. Finally, vesicle docking and fusion are regulated by small Ras-related Rab GTPases.

As previously mentioned, eukaryotic cells post-translationally modify proteins extensively through glycosylation and disulfide bond formation before leaving the cell, and such processes only take place in the ER and the GA [35]. Since alterations in the glycosylation of proteins may affect both their subsequent transport and functionality [36-38], many authors have focused their work on analyzing the possible effects of ethanol on this process. These studies, performed mainly in the liver, conclude that chronic and acute ethanol exposure affects various glycosylation steps, resulting in the synthesis of glycoproteins with changes in the composition of their oligosaccharide chain. This phenomenon is known as microheterogeneity, whose best known exponent is carbohydrate-deficient transferrin, a microheterogeneous form of serum transferrin (Tf) that is poor in sialic acid residues, which rises in serum after excessive alcohol consumption and has been used as a biological marker to detect chronic alcoholism [39,40]. Thus in the ER, alcohol exposure reduces the amount of dolichyl-phosphate, which is rate-limiting for glycoprotein synthesis. In the GA, this treatment significantly alters the levels/activity of the main enzymes, glycosyltransferases and glycosidases, which are responsible for the terminal glycosylation process [41-43].

Some of these ethanol-induced changes in the adult liver, including protein retention in the GA, also occur in the liver of rats which were prenatally exposed to ethanol [44-46]. In addition, specific morphological and biochemical alcohol-induced alterations in fetal livers have also been observed, some of which are related to the GA and to the glycosylation process. Thus, prenatal alcohol exposure has been reported to induce a striking disorganization of the GA cisternae in about 40% hepatocytes, where the cytochemical activity of several trans-GA markers, including thiamine pyrophosphatase or acid phosphatase, considerably lowered [47]. In addition, these cells displayed a significant reduction in galactosyltransferase activity [48]. Lectin cytochemistry and lectin blotting have also demonstrated that prenatal alcohol exposure results in alterations in the oligosaccharide chain composition of glycoproteins, thus inducing glycoprotein microheterogeneity [44,48]. It is interesting to note that this effect differed in adult and neonatal rat hepatocytes, suggesting that the carbohydrate moiety of some glycoproteins in neonatal hepatocytes differs from that in adult livers [44]. Moreover, recent studies using proteomics have indicated that prenatal exposure to alcohol alters the proteome of the rat offspring liver, affecting total protein, phosphorylation and glycosylation profiles, with alterations in enzymatic activities. In addition, these rats display an altered expression and post-translational modifications of the proteins involved in the substrate metabolism, cellular stress, the cytoskeleton and protein synthesis [49].

On the other hand, different studies have shown that ethanol exposure also affects the glycosylation process in other cell types, such as central nervous system cells including neurons and astrocytes, suggesting that this deleterious effect of alcohol is not unique to the liver. Thus, chronic ethanol has been reported to alter glycoconjugates and glycosyltransferases of the rat brain [50]. Studies on brain clusterin in rats have indicated that chronic ethanol treatment affects the glycosylation of this protein, which plays an important role in synaptic remodeling through its cell adhesion property or the lipid transport capacity in the brain. Ethanol treatment resulted in a lower sialation index of brain clusterin, which was accompanied by diminished activities of brain sialyltransferases and in increased activities of sialidases [51]. Along these lines, other studies have clearly shown that chronic ethanol induces the deglycosylation of brain gangliosides, which is partly due to a specific up-regulation of PM sialidase in the myelin and synaptosomal membranes in the brain [52].

Studies carried out on rat hippocampal neurons in primary culture during the proliferation and differentiation periods have revealed that chronic ethanol exposure decreases the uptake of mannose (Man), one of the sugar precursors during *N*-linked glycosylation, and alters the carbohydrate moiety of several proteins. In addition, alcohol treatment results in an increment of cell surface glycoconjugates containing terminal nonreduced Man [15]. This is an interesting result because it has been proposed that cell surface carbohydrates play critical roles in axon guidance and targeting [53,54]. Moreover, these glycans are essential in synapses, and for neuronal and postnatal viability in mice, whereas complex N-glycans seem to be dispensable in this cell type [54].

On the other hand, ethanol exposure induces the retention of glycoproteins in growing astrocytes in primary culture [20,55] and, as in neurons, long-term exposure to low doses of alcohol (30 mM) significantly affects the glycosylation in these cells. Thus, ethanol increases the uptake of several monosaccharides, which participate in glycosylation, and the extent of this effect appears to depend on the type of monosaccharide. Alcohol particularly increases the uptake of Man, *N*-acetyl-D-mannosamine, 2-deoxyglucose (2-DGlc) and glucose (Glc). An increase in Glc and 2-DGlc uptake was accompanied by an increased amount of GLUT1, the transporter of Glc in astrocytes. This treatment also affects the biosynthesis of glycoproteins, induces microheterogeneity and increases the amount of cell surface glycoproteins showing terminal Man [19]. Thus, alcohol exposure decreases not only the activity of sialyltransferase, but the levels of mannosidase II, but also increases those of sialidase in astrocytes [20,55]. One interesting finding is that alcohol exposure induces dramatic changes in the morphology and positioning of the GA of cultured astrocytes. This effect is dose-dependent and consists in both an increase of the total area occupied by the GA and a swelling of the cisternae [20].

It is unknown whether these effects of ethanol on the biosynthesis of glycoproteins could contribute, and in to what extent, to the retention of proteins as observed in both fetal and adult livers, and also in astrocytes after ethanol exposure. Since glycosylation is important for the folding of some eukaryotic proteins, alcohol-induced alterations of protein glycosylation can modify the folding of a newly synthesized protein, and thus compromise its transport [36-38,56]. Therefore, the reduced secretion rate of several glycoproteins by being exposed to ethanol may relate to the ethanol-mediated defective glycosylation of these glycoproteins. In addition, glycoproteins, such as adhesion molecules and growth factors, participate in regulating nervous system development. Thus, any alterations in the glycosylation process induced by ethanol could be a key mechanism involved in the teratogenic effects of alcohol exposure on brain development.

### 2.3. Ethanol Exposure Affects Several Factors Involved in the Transport of Nascent Proteins from the ER to the GA, as well as the Traffic Through this to the TGN

As previously stated, acute and chronic ethanol exposure induces the retention of the proteins in the GA in several cell types, including hepatocytes and astrocytes. Apart from the aforementioned alterations in the glycosylation process, other mechanisms can contribute to the retention of the proteins in the GA, which include alcohol-induced effects on the factors involved in the transport between the ER and the TGN [30]. Long-term ethanol exposure markedly reduces the vesicles that bud off the GA cisternae in the adult liver [57]. Dynamin is a GTPase which is principally involved in the scission of newly formed vesicles from the membrane of one cellular compartment, including the GA; indeed it has been proposed that the reduced budding of the vesicles from the GA in alcohol-exposed hepatocytes could be due to an increased association of dynamin with the membrane vesicles, thus delaying their release [57]. It is interesting to note that this effect was accompanied by alterations in the distribution of Rab2, which is required for the protein transport from the ER to the GA [33]. Moreover, it has been suggested that the impaired trafficking of newly synthesized O- and N-glycoslylated proteins induced by ethanol in hepatocytes is caused by the altered lipidation of Rabs, possibly Rab 1, 2, or 6 [58]. As discussed above, prenatal exposure to ethanol induces the retention of nascent proteins, which leads to an increase in the total liver protein content [43,48]. However, it is not clear whether this retention in the fetal liver affects all newly synthesized proteins, or only some of them. Unlike the adult liver, studies using albumin and Tf have indicated that prenatal alcohol exposure delays the post-GA transport of the first protein, but not of the second one [44].

On the other hand, alcohol delays the sorting from the GA of the temperature-sensitive, thermo-reversible folding mutant of VSV glycoprotein (ts045 G) in growing astrocytes in primary culture [20]. In these cells, alcohol treatment also affects several proteins involved in the ER to TGN traffic. Thus, long-term exposure to ethanol affects some SNAREs involved in the transport from the ER to the GA (rbet1 and rsec22), indicating that pre-GA transport may be affected particularly by alcohol. Moreover, alcohol lowers the expression levels of Rab GTPases Rab1, Rab2, and Rab8. Of these, Rab1 and Rab2 are implicated in the transport from the ER to the GA, whereas Rab8 is involved in post-Golgi transport. As in adult hepatocytes, alcohol exposure reduces the number of the secretory vesicles in astrocytes [20].

### 2.4. Effects of Alcohol Exposure on the Cytoskeleton

The cytoskeleton, which consists of a network of protein filaments extending throughout the cytoplasm of all eukaryotic cells, provides a structural framework for the cell, and acts as a scaffold that determines cell shape and general cytoplasm organization. In addition, the cytoskeleton is responsible for cell movements [59]. These include not only the movements of entire cells, but also the internal transport of organelles and other structures such as secretory vesicles or mitotic chromosomes through the cytoplasm. The cytoskeleton provides filamentous tracks along which membrane transport carriers are guided as they move from one compartment to another [1,4]. It is a dynamic structure that is continually reorganized and is composed of three principal types of protein filaments: microfilaments or actin filaments, IF, and MTs [59]. Both MTs and actin filaments exist in a true state of dynamic equilibrium, and are made up of subunits that can be quickly polymerized or depolymerized wherever and whenever needed. Both the organization and dynamics of the actin cytoskeleton and MTs are critical for the correct development and function of cells, and are regulated by several GTPases of the Rho family, of which the best studied are RhoA, Rac1, and Cdc42. These GTPAses regulate actin rearrangement, including the formation of lamellipodia (*via* Rac), filopodia (*via* Cdc42) and stress fibers (*via* RhoA). In addition, their activity is modulated by several regulator and effector molecules [60,61]. Efficient long distance transport within the cell is dependent on MTs. The actin cytoskeleton also plays an important role in membrane trafficking. In addition, some membrane carriers utilize both MTs and actin cytoskeletons in a single journey. Cargo transport along MTs or microfilaments is associated with a number of motor proteins, such as the MT associated motors dynein and kinesin, or the actin motor myosin [1,4].

Several studies performed in the liver have indicated that alcohol may alter the cytoskeleton, mainly MTs which would have a significant influence on intracellular trafficking and on the retention of those proteins described above [30,62]. Acetaldehyde, the first product of ethanol metabolism, forms covalent adducts with several proteins, including tubulin, after chronic ethanol exposure. The most important consequence of tubulin acetylation in the liver is that its polymerization is impaired. However, it hyperstabilizes once MTs have formed. Therefore, increased acetylation and stability of MTs explain, in part, the alcohol-induced defects in protein trafficking [30,62,63]. This effect may also occur in the hepatocytes of those rats prenatally exposed to ethanol, where this toxin increases tubulin content and diminishes in vitro MT's polymerization [44]. In addition, whereas alcohol exposure increases the total protein levels of Rac and Cdc42, the activated GTP-bound forms of these two GTPases significantly decrease in the presence of ethanol. These effects are accompanied by a lack of effect of ethanol on either RhoA activation or the protein expression levels, which may be due to the absence of stress fibers in hepatocytes [62].

Chronic alcohol exposure (30-100 mM) dramatically affects the three constituents of the cytoskeleton (actin filaments, IF and MTs) in astrocytes in primary culture during the proliferation and differentiation periods [20,64-68]. Of these constituents, alcohol modifies the organization and function of the two components involved in the traffic, i.e., actin and MTs. Thus, in relation to actin, alcohol induces the dissolution of stress fibers and the appearance of circular filaments beneath the PM [18,64,66,68]. It is interesting to observe that this effect is reversible when astrocytes are incubated with lysophosphatidic acid [68], which is a potent lipid mediator with a growth factor and actin rearrangement activities. Moreover, chronic ethanol treatment of rat astrocytes specifically lowers the endogenous levels of active RhoA, which is closely related with stress fibers formation. Furthermore, alcohol exposure not only affects the MTs' polymerization in these cells, but reduces the expression levels of motor proteins kinesin and dynein, which play important roles in post-GA trafficking. In addition, the structure of the GA and its function depends to large extent on the integrity of MTs and actin filaments [69,70]. Therefore, the alterations induced by ethanol to these cytoskeleton elements may be involved in the retention of proteins in the GA of ethanol-exposed astrocytes.

The analysis of the effect of chronic alcohol on the neuronal cytoskeleton reveals important changes in the organization and function of MTs and microfilaments [71,72]. In addition, these changes are accompanied by changes in the levels of several molecules associated with the organization and dynamics of both the cytoskeleton components. Thus, the amount of both polymerized tubulin and filamentous actin decreases in cultured hippocampal neurons exposed to alcohol. This treatment also lowers the number of MTs in dendrites, and diminishes the MT-associated protein-2 (MAP2) levels, which mainly localizes in the somatodendritic compartment in neurons. However, there is no information available about the effect that alcohol has on motor proteins such as KIFs (kinesin superfamily proteins) or dynein in neurons. An alteration in these molecules' levels or function could explain the effect of alcohol exposure on the axonal transport of different cargoes in the peripheral nervous system [73-75]. It is noteworthy that alcohol exposure does not change the acetylated tubulin levels in neurons, suggesting that acetylation does not apparently play an important role in alcohol-induced alterations to neuronal MTs [71]. On the other hand, ethanol lowers the levels of total Rac, Cdc42, and RhoA. However, although alcohol lowers the levels of the active forms (GTP bound) of Rac1 and Cdc42, it does not affect the active form of RhoA. Finally, the levels of several regulator and effectors molecules of these GTPases change with heterogeneous results.

### 2.5. Ethanol Affects Endocytosis

Endocytosis is a process that plays key roles in the positive regulation of many intracellular signaling cascades. Through endocytosis, cells internalize different types of molecules, including nutrients, ligands, and PM proteins and lipids from the cell surface [76-78]. Once internalized, the membrane and content of the resulting endosome can be recycled back out to the PM, or be addressed through the late endosome to lysosomes, where they are degraded. In addition to the well-known clathrin-dependent mechanism of endocytosis, several pathways that do not use a clathrin coat are described. Thus, endocytosis can occur through structures coated with the caveolin protein. Moreover, there are other endocytosis types which are clathrin- and caveolin-independent. However, these pathways may share some of the same molecular machinery, especially that related to actin polymerization in membrane remodeling. The effect of alcohol on endocytosis has been widely studied in hepatic cells, and there are reports that whereas clathrin-mediated endocytosis is selectively impaired by ethanol, the internalization of the markers for caveolae/raft-mediated endocytosis, fluid phase internalization or nonvesicle-mediated uptake was not impaired in ethanol-treated cells [30,79]. This effect has been attributed to a deleterious effect of ethanol exposure on the vesicle fission from the plasma membrane [79].

Studies that have analyzed the effect of chronic exposure to ethanol on endocytosis in astrocytes in primary culture indicate that this treatment affects clathrin-mediated endocytosis, caveolin-mediated endocytosis and fluid phase internalization, respectively revealed by Tf, bovine serum albumin (BSA) and horseradish peroxidase used as markers. Moreover, ethanol exposure also induces a fragmentation of the tubular endosomes in these cells, and also lowers the levels of clathrin, caveolin, and of Rab5, a small GTPase localized to early endosomes which regulates the fusion between endocytic vesicles and early endosomes, as well as the homotypic fusion between early endosomes [80]. Sphingolipids are PM components, and are particularly abundant in the central nervous system where they are enriched in the caveolae [78]. They are transported along the exocytic and endocytic pathways in eukaryotic cells. Like proteins, after endocytosis, sphingolipids are sorted to distinct intracellular organelles prior to recycling or degradation. In astrocytes in primary culture, endocytosed plasma membranes C_6_-NBD-glucosylceramide (NBD-GlcCer) and C_6_-NBD-sphingomyelin (NBD-SM) are diverted to the GA and lysosomes, respectively. Chronic ethanol exposure does not alter this endocytic sorting, but delays the internalization of both NBD-sphingolipids. Moreover, ethanol also stimulates the in situ metabolism of NBD-ceramide to NBD-GlcCer and NBD-SM. Thus, exposure to chronic ethanol perturbs the lipid endocytic process and stimulates the de novo synthesis of NBD-sphingolipids by shifting the balance of sphingolipid metabolism in favor of the sphingomyelin pathway [81]. Moreover, recent studies have indicated that chronic alcohol exposure also reduces intracellular ionic zinc levels and extracellular zinc uptake, resulting in poorer zincosome formation. Given the endocytic nature of zincosomes, the effect of ethanol on endocytosis is apparently the origin of this deficit [82].

In addition, exposure of neurons in primary culture to chronic ethanol affects clathrin-dependent and clathrin-independent endocytosis as it probably acts not only on the various proteins directly involved in the several steps of these processes, but also on the proteins participating in the organization and dynamics of the actin cytoskeleton, as mentioned before. In addition to RhoGTPases RhoA, Rac1 and Cdc42, these proteins include clathrin, adaptor AP-2, sorting nexin SNX9, GTPases Rab5, Rab11, or endosomal marker EEA1. In addition, ethanol exposure also affects the cholesterol cellular levels related with clathrin-independent endocytosis [17]. A toxic effect of alcohol on endocytosis can affect some important neuronal activities that depend on the endocytic process, including synaptic vesicle recycling, regulation of the number of signaling receptors, trafficking of postsynaptic receptors, polarized axon elongation, growth cone navigation, neuronal migration and hippocampal plasticity. Thus, for example, ethanol alters the endosomal recycling of the dopamine transporter, responsible for terminating dopamine signaling by rapidly removing the transmitter from the synaptic cleft region [83]. Alcohol treatment has also shown to affect the *N*-methyl-D-aspartate receptor function [84] and the release of different transmitters from synaptic terminals [85].

### 2.6. Alcohol-Induced Alterations in Nucleocytoplasmic Shuttle

The proteins synthesized in the cytoplasm can be targeted to a number of distinct cell compartments, including the nucleus which is separated from the cytoplasm by the nuclear envelope (NE). The NE is penetrated by nuclear pore complexes (NPCs) that allow the exchange of macromolecules between the two compartments [86]. Protein trafficking into and out of the nucleus is known as nucleocytoplasmic transport, and the continuous movement back and forth across the NE is termed nucleocytoplasmic shuttling [86,87,88]. Nucleocytoplasmic transport is a complex process that comprises a multitude of substrates, and which occurs in several steps: a) recognition of the cargo import/export signal by an import or export receptor [importins (Imp)/exportins (Exp)]; b) docking of the cargo/receptor assembly at the NPC; c) translocation across the NPC; d) release of the transported protein; and e) recycling of the receptors for the next round of transport. Nucleocytoplasmic transport not only serves to enable the operation of the basal replication, transcription and processing machinery, but to also regulate the cell cycle, transcriptional activation and repression, circadian rhythms, cell differentiation, as well as a whole host of other essential processes [89,90].

Despite the importance of nucleocytoplasmic transport, there is very little information about the possible effects of alcohol exposure on this transport, and the bulk of this information has been obtained from astrocytes in primary culture [16]. In these cells, chronic ethanol exposure has been shown to induce a significant delay of both the import and export of proteins to the nucleus. It is noteworthy to mention that whereas this treatment affects neither the amount nor the distribution of several representative proteins participating in nuclear import, such as RanGAP1, Ran-BP1, and importins α2 and β3, it diminishes the amount of Exp1/CRM1, a general export receptor involved in nuclear export. An important finding is that ethanol exposure does not equally affect all the proteins transported between the cytoplasm and the nucleus. Moreover, ethanol also affects the number and distribution of NPCs; in the former, alcohol increases the total number of pores; in the latter case, ethanol exposure results in a change from a random distribution pattern to another clumped one. These changes may relate to changes in either the levels of several proteins which participate in NPC composition, such as RanBP2 or Nup358, and nucleoporin p62, or the amount of lamins which perform important functions in positioning NPCs in the NE. In contrast to these results in astrocytes, other studies have reported that long-term alcohol-fed rats show a marked reduction in the total number of NPCs in rat hippocampal neurons [91].

Although there is no information available on the role of nucleocytoplasm transport in relation to astrocytes' specific functions, it is known that this process is fundamental in regulating the diverse cellular activities in most cells, including growth factor-mediated signaling, stress responses, cell cycle control, cell death by apoptosis, development, gene transcription and translation. Moreover, the NPC is currently emerging as an important regulator of gene expression given its influence on the internal architectural organization of the nucleus and its apparently extensive involvement in coordinating the seamless delivery of genetic information to cytoplasmic protein synthesis machinery [92]. Many of these activities, which are also present in astrocytes, are altered by chronic ethanol exposure in these cells and in others.

## 3. Conclusions

Different studies have mainly indicated that moderate ethanol exposure impairs intracellular traffic (exocytosis, endocytosis and nucleocytoplasmic transport) in several cell types, including central nervous system cells, indicating that this process is a target for the toxic effects of ethanol in these cells. This may be caused by the deleterious effects of ethanol on several of the processes involved in the biosynthesis of glycoconjugates, including the regulation of ER to GA transport carriers, the glycosylation process, the GA's morphological and functional properties, and the MT's and the actin cytoskeleton's activities. It is not uncommon to observe slight effects in chronic treatments with low doses of alcohol. Nevertheless, while most cellular machinery remains functional under such conditions, it works less effectively or more slowly. This may be significant in processes such as neuronal migration where temporal regulation is fundamental.

## Abbreviations

2-DGlc: 2-deoxyglucose
BSA: bovine serum albumin
ER: endoplasmic reticulum
FAS: fetal alcoholic syndrome
GA: Golgi apparatus
Glc: glucose
IF: intermediate filaments
Man: Mannose
MT: microtubules
NE: nuclear envelope
NPC: nuclear pore complex
PM: plasma membrane
Tf: transferrin
TGN: trans-Golgi network

## References

[1] Derby M.C., Gleeson P.A. (2007). New insights into membrane trafficking and protein sorting. Int. Rev. Cytol..

[2] Jackson C.L. (2009). Mechanisms of transport through the Golgi complex. J. Cell Sci..

[3] Stephens D.J., Pepperkok R. (2002). Imaging of procollagen transport reveals COPI-dependent cargo sorting during ER-to-Golgi transport in mammalian cells. J. Cell Sci..

[4] van Vliet C., Thomas E.C., Merino-Trigo A., Teasdale R.D., Gleeson P.A. (2003). Intracellular sorting and transport of proteins. Prog. Biophys. Mol. Biol..

[5] Hua Z., Graham T.R., Segev N. (2009). The Golgi apparatus. Trafficking Inside Cells: Pathways, Mechanisms and Regulation.

[6] Roth J. (2002). Protein n-glycosylation along the secretory pathway: Relationship to organelle topography and function, protein quality control, and cell interactions. Chem. Rev..

[7] Spiro R.G. (2002). Protein glycosylation: Nature, distribution, enzymatic formation, and disease implications of glycopeptide bonds. Glycobiology.

[8] Room R., Babor T., Rehm J. (2005). Alcohol and public health. Lancet.

[9] Goodlett C.R., Horn K.H. (2001). Mechanisms of alcohol-induced damage to the developing nervous system. Alcohol Res. Health.

[10] Streissguth A.P., O'Malley K. (2000). Neuropsychiatric implications and long-term consequences of fetal alcohol spectrum disorders. Semin. Clin. Neuropsychiatry.

[11] Sokol R.J., Delaney-Black V., Nordstrom B. (2003). Fetal alcohol spectrum disorder. JAMA.

[12] May P.A., Gossage J.P., Kalberg W.O., Robinson L.K., Buckley D., Manning M., Hoyme H.E. (2009). Prevalence and epidemiologic characteristics of FASD from various research methods with an emphasis on recent in-school studies. Dev. Disabil. Res. Rev..

[13] Guerri C., Bazinet A., Riley E.P. (2009). Fetal alcohol spectrum disorders and alterations in brain and behaviour. Alcohol Alcohol..

[14] Martínez S.E., Egea G. (2007). Novel molecular targets for the prevention of fetal alcohol syndrome. Recent Pat. CNS Drug Discov..

[15] Braza-Boils A., Tomás M., Marín M.P., Megías L., Sancho-Tello M., Fornas E., Renau-Piqueras J. (2006). Glycosylation is altered by ethanol in rat hippocampal cultures neurons. Alcohol Alcohol..

[16] Marín M.P., Tomás M., Esteban-Pretel G., Megías L., López-Iglesias C., Egea G., Renau-Piqueras J. (2008). Chronic ethanol exposure induces alterations in the nucleocytoplasmic transport in growing astrocytes. J. Neurochem..

[17] Marín M.P., Esteban-Pretel G., Ponsoda X., Romero A.M., Ballestín R., López C., Megías L., Timoneda T., Molowny A., Canales J.J. (2010). Endocytosis is altered by chronic alcohol exposure in cultured neurons. Toxicol. Sci..

[18] Martínez S., Lázaro-Diéguez F., Selva J., Clavo F., Renau-Piqueras J., Crespo P., Claro E., Egea G. (2007). Lysophosphatidic acid rescues RhoA activation and phosphoinositides levels in astrocytes exposed to ethanol. J. Neurochem..

[19] Tomás M., Fornas E., Megías L., Durán J.M., Portolés M., Guerri C., Egea G., Renau-Piqueras J. (2002). Ethanol impairs monosaccharide uptake and glycosylation in cultured rat astrocytes. J. Neurochem..

[20] Tomás M., Marín P., Megías L., Egea G., Renau-Piqueras J. (2005). Ethanol perturbs the secretory pathway in astrocytes. Neurobiol. Dis..

[21] Karinch A.M., Martin J.H., Vary T.C. (2008). Acute and chronic ethanol consumption differentially impact pathways limiting hepatic protein synthesis. Am. J. Physiol. Endocrinol. Metab..

[22] Vallés S., Lindo L., Montolíu C., Renau-Piqueras J., Guerri C. (1994). Prenatal exposure to ethanol induces changes in the nerve growth factor and its receptor in proliferating astrocytes in primary culture. Brain Res..

[23] Miñana R., Climent E., Barettino D., Segui J.M., Renau-Piqueras J., Guerri C. (2000). Alcohol exposure alters the expression pattern of neural cell adhesion molecules during brain development. J. Neurochem..

[24] Heaton M.B., Madorsky I., Paiva M., Siler-Marsiglio K.I. (2004). Ethanol-induced reduction of neurotrophin secretion in neonatal rat cerebellar granule cells is mitigated by vitamin E. Neurosci. Lett..

[25] Davis M.I. (2008). Ethanol-BDNF interactions: still more questions than answers. Pharmacol. Ther..

[26] Imperato A., di Chiara G. (1986). Preferential stimulation of dopamine release in the nucleus accumbens of freely moving rats by ethanol. J. Pharmacol. Exp. Ther..

[27] Luo J., Miller M.W. (1999). Transforming growth factor beta1-regulated cell proliferation and expression of neural cell adhesion molecule in B104 neuroblastoma cells: differential effects of ethanol. J. Neurochem..

[28] Baraona E. (1975). Alcoholic hepatomegaly: accumulation of proteins in the liver. Science.

[29] Baraona E., Leo M., Borowsky S., Lieber C. (1977). Pathogenesis of alcohol-induced accumulation of protein in the liver. J. Clinic. Invest..

[30] Shepard B.D., Fernandez D.J., Tuma P.L. (2010). Alcohol consumption impairs hepatic protein trafficking: mechanisms and consequences. Genes Nutr..

[31] Stein O., Stein Y. (1966). Visualization of intravenously injected 9, 10-3-H2-palmitic acid in rat liver by electronmicroscopic autoradiography. Isr. J. Med. Sci..

[32] Stein O., Stein Y. (1967). Lipid synthesis, intracellular transport, storage, and secretion. Electron microscopic radioautographic study of liver after injection of tritiated palmitate or glycerol in fasted and ethanol-treated rats. J. Cell Biol..

[33] Larkin J.M., Oswald B., McNiven M.A. (1996). Ethanol-induced retention of nascent proteins in rat hepatocytes is accompanied by altered distribution of the small GTP-binding protein Rab2. J. Clin. Invest..

[34] Ehrenreich J.H., Bergeron J.J., Siekevitz P., Palade G.E. (1973). Golgi fractions prepared from rat liver homogenates. Isolation procedure and morphological characterization. J. Cell Biol..

[35] Schwartz T.U., Jékely G. (2007). Origins and evolution of cotranslational transport to the ER. Eukaryotic Membranes and Cytoskeleton: Origins and Evolution.

[36] Mitra N., Sinha S., Ramya T.N., Surolia A. (2006). N-linked oligosaccharides as outfitters for glycoprotein folding, form and function. Trends Biochem. Sci..

[37] Rasmussen J.R. (1992). Effect of glycosylation on protein function. Curr. Opin. Struct. Biol..

[38] Shental-Bechor D., Levy Y. (2009). Folding of glycoproteins: Toward understanding the biophysics of the glycosylation code. Curr. Opin. Struct. Biol..

[39] Arndt T. (2001). Carbohydrate-deficient transferrin as a marker of chronic alcohol abuse: A critical review of preanalysis, analysis, and interpretation. Clin. Chem..

[40] Das S.K., Dhanya L., Vasudevan D.M. (2008). Biomarkers of alcoholism: An updated review. Scand. J. Clin. Lab. Invest..

[41] Cottalasso D., Gazzo P., Dapino D., Domenicotti C., Pronzato M.A., Traverso N., Bellocchio A., Nanni G., Marinari U.M. (1996). Effect of chronic ethanol consumption on glycosylation processes in rat liver microsomes and Golgi apparatus. Alcohol Alcohol..

[42] Ghosh P., Liu Q.H., Lakshman M.R. (1995). Long-term ethanol exposure impairs glycosylation of both N- and O-glycosylated proteins in rat liver. Metabolism.

[43] Guasch R., Renau-Piqueras J., Guerri C. (1992). Chronic ethanol consumption induces accumulation of proteins in the liver Golgi apparatus and decreases galactosyltransferase activity. Alcohol. Clin. Exp. Res..

[44] Azorín I., Portolés M., Marín P., Lázaro-Diéguez F., Megías L., Egea G., Renau-Piqueras J. (2004). Prenatal ethanol exposure alters the cytoskeleton and induces glycoprotein microheterogeneity in rat newborn hepatocytes. Alcohol Alcohol..

[45] Renau-Piqueras J., Guerri C., Sancho-Tello M., Báguena-Cervellera R., Miragall F. (1989). Effects of prenatal exposure to ethanol on rat liver development. A review. Int. J. Dev. Biol..

[46] Renau-Piqueras J., Sancho-Tello J., Báguena-Cervellera R., Guerri C. (1989). Prenatal exposure to ethanol alters the synthesis and glycosylation of proteins in fetal hepatocytes. Alcohol. Clin. Exp. Res..

[47] Renau-Piqueras J., Miragall F., Guerri C., Báguena-Cervellera R. (1987). Prenatal exposure to alcohol alters the Golgi apparatus of newborn rat hepatocytes. A cytochemical study. J. Histochem. Cytochem..

[48] Renau-Piqueras J., Guasch R., Azorín I., Seguí J.M., Guerri C. (1997). Prenatal ethanol exposure affects galactosyltransferase activity and glycoconjugates in the Golgi apparatus of fetal rat hepatocytes. Hepatology.

[49] Fofana B., Yao X.H., Rampitsch C., Cloutier S., Wilkins J.A., Nyomba B.L. (2010). Prenatal alcohol exposure alters phosphorylation and glycosylation of proteins in rat offspring liver. Proteomics.

[50] Omodeo-Salé F., Palestini P. (1994). Chronic ethanol effects on glycoconjugates and glycosyl-transferases of rat brain. Alcohol.

[51] Hale E.A., Raza S.K., Ciecierski R.G., Ghosh P. (1998). Deleterious actions of chronic ethanol treatment on the glycosylation of rat brain clusterin. Brain Res..

[52] Azuine M.A., Patel S.J., Lakshman M.R. (2006). Effects of chronic ethanol administration on the activities and relative synthetic rates of myelin and synaptosomal plasma membrane-associated sialidase in the rat brain. Neurochem. Int..

[53] Lipscomb B.W., Treloar H.B., Greer C.A. (2002). Cell surface carbohydrates reveal heterogeneity in olfactory receptor cell axons in the mouse. Cell Tissue Res..

[54] Ye Z., Marth J.D. (2004). N-glycan branching requirement in neuronal and postnatal viability. Glycobiology.

[55] Miñana R., Climent E., Barettino D., Segui J.M., Renau-Piqueras J., Guerri C. (2000). Alcohol exposure alters the expression pattern of neural cell adhesion molecules during brain development. J. Neurochem..

[56] Roth J., Zuber C., Park S., Jang I., Lee Y., Kysela K.G., Le Fourn V., Santimaria R., Guhl B., Cho J.W. (2010). Protein N-glycosylation, protein folding, and protein quality control. Mol. Cells.

[57] Török N., Marks D., Hsiao K., Oswald B.J., McNiven M.A. (1997). Vesicle movement in rat hepatocytes is reduced by ethanol exposure: alterations in microtubule-based motor enzymes. Gastroenterology.

[58] Marmillot P., Rao M.N., Lakshman M.R. (2001). Chronic ethanol exposure in rats affects rabs-dependent hepatic trafficking of apolipoprotein E and transferrin. Alcohol.

[59] Fletcher D.A., Mullins R.D. (2010). Cell mechanics and the cytoskeleton. Nature.

[60] Iden S., Collard J.G. (2008). Crosstalk between small GTPases and polarity proteins in cell polarization. Nat. Rev. Mol. Cell Biol..

[61] Govek E.E., Newey S.E., van Aelst L. (2005). The role of the Rho GTPases in neuronal development. Genes Dev..

[62] Shepard B.D., Tuma P.L. (2010). Alcohol-induced alterations of the hepatocyte cytoskeleton. World J. Gastroenterol..

[63] Yoon Y., Török N., Krueger E., Oswald B., McNiven M.A. (1998). Ethanol-induced alterations of the microtubule cytoskeleton in hepatocytes. Am. J. Physiol..

[64] Allansson L., Khatibi S., Olsson T., Hansson E. (2001). Acute ethanol exposure induces (Ca^2+^) transients, cell swelling and transformation of actin cytoskeleton in astroglial primary cultures. J. Neurochem..

[65] Gonzalez A., Salido G.M. (2009). Ethanol alters the physiology of neuron-glia communication. Int. Rev. Neurobiol..

[66] Guasch R.M., Tomás M., Minambres R., Valles S., Renau-Piqueras J., Guerri C. (2003). RhoA and lysophosphatidic acid are involved in the actin cytoskeleton reorganization of astrocytes exposed to ethanol. J. Neurosci. Res..

[67] Renau-Piqueras J., Zaragozá R., de Paz P., Báguena-Cervellera R., Megías L., Guerri C. (1989). Effects of prolonged ethanol exposure on the glial fibrillary acidic protein-containing intermediate filaments of astrocytes in primary culture: A quantitative immunofluorescence and immunogold electron microscopic study. J. Histochem. Cytochem..

[68] Tomás M., Lázaro-Diéguez F., Durán J.M., Marín P., Renau-Piqueras J., Egea G. (2003). Protective effects of lysophosphatidic acid (LPA) on chronic ethanol-induced injuries to the cytoskeleton and on glucose uptake in rat astrocytes. J. Neurochem..

[69] Egea G., Lázaro-Dieguéz F., Vilella M. (2006). Actin dynamics at the Golgi complex in mammalian cells. Curr. Opini. Cell Biol..

[70] Egea G., Ríos R.M., Minorov A., Pavelka M. (2008). The role of the cytoskeleton in the structure and function of the Golgi apparatus. The Golgi Apparatus. State of the Art 110 years after Camilo Golgi's discovery.

[71] Romero A.M., Esteban-Pretel G., Marín M.P., Ponsoda X., Ballestín R., Canales J.J., Renau-Piqueras J. (2010). Chronic ethanol exposure alters the levels, assembly and cellular organization of the actin cytoskeleton and microtubules in hippocampal neurons in primary culture. Toxicol. Sci..

[72] Sordella R., van Aelst L. (2006). Driving actin dynamics under the influence of alcohol. Cell.

[73] McLane J.A. (1990). Retrograde axonal transport in chronic ethanol-fed and thiamine-deficient rats. Alcohol..

[74] McLane J.A., Atkinson M.B., McNulty J., Breuer A.C. (1992). Direct measurement of fast axonal organelle transport in chronic ethanol-fed rats. Alcohol Clin. Exp. Res..

[75] Koike H., Sobue G. (2006). Alcoholic neuropathy. Curr. Opin. Neurol..

[76] Doherty G.J., McMahon H.T. (2009). Mechanisms of endocytosis. Annu. Rev. Biochem..

[77] Donaldson J.G., Porat-Shliom N., Cohen L.A. (2009). Clathrin-independent endocytosis: A unique platform for cell signalling and PM remodeling. Cell. Signal..

[78] Mayor S., Pagano R.E. (2007). Pathways of clathrin-independent endocytosis. Nat. Rev. Mol. Cell Biol..

[79] Fernandez D.J., McVicker B.L., Tuma D.J., Tuma P.L. (2009). Ethanol selectively impairs clathrin-mediated internalization in polarized hepatic cells. Biochem. Pharmacol..

[80] Megías L., Guerri C., Fornas E., Azorín I., Bendala E., Sancho-Tello M., Durán J.M., Tomás M., Gomez-Lechón M.J., Renau-Piqueras J. (2000). Endocytosis and transcytosis in growing astrocytes in primary culture. Effect of ethanol and possible implications in neural development. Int. J. Dev. Biol..

[81] Tomás M., Durán J.M., Lázaro-Diéguez F., Babia T., Renau-Piqueras J., Egea G. (2004). Fluorescent analogues of plasma membrane sphingolipids are sorted to different intracellular compartments in astrocytes. Harmful effects of chronic ethanol exposure on sphingolipid trafficking and metabolism. FEBS Lett..

[82] Ballestín R., Molowny A., Marín M.P., Esteban-Pretel G., Romero A.M., López-García C., Renau-Piqueras J., Ponsoda X. (2011). Ethanol reduces zincosome formation in cultured astrocytes. Alcohol Alcohol..

[83] Methner N.R., Dayne Mayfield R. (2010). Ethanol alters endosomal recycling of human dopamine transporters. J. Biol. Chem..

[84] Hicklin T.R., Wu P.H., Radcliffe R.A., Freund R.K., Goebel-Goody S.M., Correa P.R., Proctor W.R., Lombroso P.J., Browning M.D. (2011). Alcohol inhibition of the NMDA receptor function, long-term potentiation, and fear learning requires striatal-enriched protein tyrosine phosphatase. Proc Natl Acad Sci U.S.A..

[85] Roberto M., Treistman S.N., Pietrzykowski A.Z., Weiner J., Galindo R., Mameli M., Valenzuela F., Zhu P.J., Lovinger D., Zhang T.A. (2006). Actions of acute and chronic ethanol on presynaptic terminals. Alcohol Clin. Exp. Res..

[86] Wente S.R., Rout M.P. (2010). The nuclear pore complex and nuclear transport. Cold Spring Harb. Perspect. Biol..

[87] Köser J., Maco B., Aebi U., Fahrenkrog B. (2005). The nuclear pore complex becomes alive: New insights into its dynamics and involvement in different cellular processes. Atlas Genet. Cytogenet. Oncol. Haematol..

[88] Michel W.M. (2000). Nucleocytoplasmic shuttling signals: Two for the price of one. Trends Cell Biol..

[89] Macara I.G. (2000). Transport into and out of the nucleus. Microbiol. Mol. Biol. Rev..

[90] Yoneda Y. (2001). Nucleocytoplasmic protein traffic and its significance to cell function. Genes Cells.

[91] Andrade J.P., Cadete-Leite A., Paula-Barbosa M.M., Volk B., Tavares M.A. (1988). Long-term alcohol consumption reduces the number of neuronal nuclear pores. A morphometric study undertaken in CA3 hippocampal pyramids of rats. Alcohol Clin. Exp. Res..

[92] Strambio-De-Castillia C., Niepel M., Rout M.P. (2010). The nuclear pore complex: Bridging nuclear transport and gene regulation. Nat. Rev. Mol. Cell Biol..

